# Amyloid β-induced impairments on mitochondrial dynamics, hippocampal neurogenesis, and memory are restored by phosphodiesterase 7 inhibition

**DOI:** 10.1186/s13195-018-0352-4

**Published:** 2018-02-20

**Authors:** Fernando Bartolome, Macarena de la Cueva, Consuelo Pascual, Desiree Antequera, Tamara Fernandez, Carmen Gil, Ana Martinez, Eva Carro

**Affiliations:** 1 Group of Neurodegenerative Diseases, Hospital 12 de Octubre Research Institute (imas12), 28041 Madrid, Spain; 2 Networked Biomedical Research Center in Neurodegenerative Diseases (CIBERNED), 28031 Madrid, Spain; 3 Centro de Investigaciones Biológicas-CSIC, Ramiro de Maeztu 9, 28040 Madrid, Spain

**Keywords:** Phosphodiesterase, Alzheimer’s disease, Transgenic mice, Oral administration, Memory, Hippocampus, Neurogenesis, Mitochondria, Mitophagy

## Abstract

**Background:**

The phosphodiesterase (PDE) 7 inhibitor S14 is a cell-permeable small heterocyclic molecule that is able to cross the blood–brain barrier. We previously found that intraperitoneal treatment with S14 exerted neuroprotection in an Alzheimer’s disease (AD) model (in APP/PS1 mice). The objective of this study was to investigate the neurogenic and cellular effects of oral administration of S14 on amyloid β (Aβ) overload.

**Methods:**

We orally administered the PDE7 inhibitor S14 (15 mg/kg/day) or vehicle in 6-month-old APP/PS1 mice. After 5 weeks of S14 treatment, we evaluated cognitive functions and brain tissues. We also assessed the effects of S14 on the Aβ-treated human neuroblastome SH-SY5Y cell line.

**Results:**

Targeting the cyclic adenosine monophosphate (cAMP)/cAMP-response element binding protein (CREB) pathway, S14 rescued cognitive decline by improving hippocampal neurogenesis in APP/PS1 transgenic mice. Additionally, S14 treatment reverted the Aβ-induced reduction in mitochondrial mass in APP/PS1 mice and in the human neuroblastoma SH-SY5Y cells co-exposed to Aβ. The restoration of the mitochondrial mass was found to be a dual effect of S14: a rescue of the mitochondrial biogenesis formerly slowed down by Aβ overload, and a reduction in the Aβ-increased mitochondrial clearance mechanism of mitophagy.

**Conclusions:**

Here, we show new therapeutic effects of the PDE7 inhibitor, confirming S14 as a potential therapeutic drug for AD.

**Electronic supplementary material:**

The online version of this article (10.1186/s13195-018-0352-4) contains supplementary material, which is available to authorized users.

## Background

Alzheimer’s disease (AD) is the most common cause of dementia and accelerates with advancing age. The most prominent symptoms of the disease are the progressive decline in cognitive functions and memory. AD is characterized by three major neuropathological hallmarks: senile plaques composed of amyloid β (Aβ) peptides, intracellular neurofibrillary tangles, and neuronal loss. The loss of neurons and synapses spreads to the hippocampus, entorhinal cortex, and frontal cortex, all of which play important roles in reference and working memory [1, 2]. In particular, the hippocampus is critical for learning and memory as well as mood regulation, and adult neurogenesis is necessary for its normal function [3, 4]. Adult hippocampal neurogenesis is known to contribute to the processing and the storage of new information [5, 6]. In AD brains the pathophysiological environment could have adverse effects on neurogenesis [7]. While memory deficits observed in AD could be linked to alterations in hippocampal neurogenesis [8, 9] we still need to actively investigate how neurogenesis is linked to cognitive function and whether stimulating regenerative mechanisms in the brain could restore or prevent further deterioration of cognition during the disease process. During the process of synaptic strengthening and memory formation a variety of molecular mechanisms are activated. The cyclic adenosine monophosphate (cAMP)/cAMP-response element binding protein (CREB) pathway may regulate the transcription of genes controlling these processes. Many neurodegenerative diseases are associated with aberrant cyclic nucleotide signalling related to phosphodiesterase (PDE) expression. PDEs are key enzymes in the cAMP signalling cascade. In AD brains, changes in cAMP-specific PDE mRNA expression were detected [10, 11]. PDE7 inhibition has been implicated in inflammation and neurodegenerative disorders [12, 13]. Peripheral administration of S14, a small heterocyclic molecule able to inhibit the cAMP-specific PDE7, produced anxiolytic-like effects, restored visual short-term memory, and decreased different pathological hallmarks in brains of APP/PS1 mice [14]. S14 treatment also induced *in vitro* and *in vivo* proliferation of neural stem cells, promoting their differentiation toward a dopaminergic phenotype in the substantia nigra of hemiparkinsonian rats [15]. More recently, the PDE7 inhibitor showed a significant increase in newly generated neurons in the olfactory bulb and the hippocampus of adult rats [16]. While PDE inhibitors have demonstrated neurotrophic, neuroprotective, and immunomodulatory potencies in AD models, little is known about the role of PDE7 in hippocampal neurogenesis in AD and its effects on learning and memory.

Through activation of peroxisome proliferator-activated receptor γ co-activator α (PGC-1α) and regulating p62 degradation, the cAMP/CREB pathway may also control mitochondrial dynamics and autophagy, respectively [17–21]. Mitochondria are important organelles in cells and more so in neurons since these demand high amounts of energy for almost all cellular processes including cell survival and death, calcium homeostasis regulation, synaptic plasticity, and neurotransmitter synthesis [22]. Mitochondrial dysfunction has been further demonstrated in the pathogenesis of neurodegenerative disorders [23–25] but not much is known about the interaction of PDE inhibition and mitochondrial dynamics in neurodegeneration.

In this study, we evaluated the effects of oral administration of S14 in APP/PS1 mice. Our results demonstrated that S14 rescued memory deficits and increased hippocampal neurogenesis mediated by upregulation of the pCREB signalling pathway. Additionally, we reported that S14 treatment restored the Aβ-induced impairments in mitochondrial dynamics in APP/PS1 mice and in the human neuroblastoma SH-SY5Y cells co-exposed to Aβ.

## Methods

### PDE inhibitor S14

S14 was synthesized in CIB-CSIC following previously described procedures [26]. The compound was diluted in 0.5% *w/v* sodium carboxyl methyl cellulose (Na-CMC) and 0.1% *v/v* Tween 80 in water suspension. Pharmacokinetic data for S14 showing a brain to plasma ratio oral bioavailability of 0.2 and the effective dose for experiments of this compound after oral administration calculated through evaluation of brain and plasma levels of S14 have been previously reported [14].

### Animal experiments

Male double transgenic APP/PS1 mice (6 months old), a cross between Tg2576 (overexpressing human APP695) and mutant PS1 (M146 L), were used from our inbred colony (Instituto de Investigacion Hospital 12 de Octubre). Age-matched mice not expressing the transgene were used as wild-type controls. The PDE7 inhibitor S14 was orally administered in a 0.5% *w/v* sodium carboxyl methyl cellulose (Na-CMC) and 0.1% *v/v* Tween 80 in water suspension. For testing the effects of oral chronic treatment on memory and neurogenesis, S14 (15 mg/kg) or vehicle was given once per day for 5 weeks. At the end of treatment, animals were deeply anaesthetised and perfused transcardially either with saline for biochemical analysis, or 4% paraformaldehyde (PFA) in 0.1 M phosphate buffer (PB), pH 7.4, for immunohistochemical analysis. All animals were handled and cared for according to the Council Directive 2010/63/UE of 22 September 2010.

### Measurement of learning and memory

**Fig. 1:**
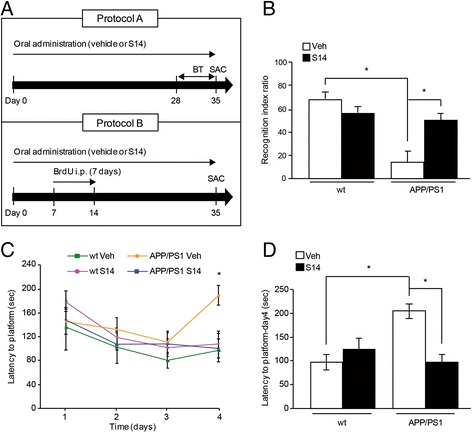
Behavioural performance of APP/PS1 and wild-type (wt) mice after oral treatment with S14. **a** Protocol A: treatment schedule for memory testing. Behavioural tests (BT) were carried out on days 28–35. S14 or vehicle (0.5% *w/v* Na-CMC and 0.1% *v/v* Tween 80 in water suspension) were orally administered once per day for 5 weeks until the end of the tests (day 35) when the animals were sacrificed (SAC). Protocol B: treatment schedule for neurogenesis testing. 5-Bromo-2’-deoxyuridine (BrdU) or saline was injected intraperitoneally (i.p.) on days 7–14. S14 or vehicle was orally administered once per day for 5 weeks until the end of the treatment (day 35) when the animals were sacrificed (SAC). **b** In the novel-object recognition test, APP/PS1 mice showed a significant decline in performance. In APP/PS1 mice orally treated with S14 for 5 weeks, the ratio exploring the novel object was enhanced. Data are expressed as mean ± SEM; *n* = 9–13 mice/group; *p < 0.05. **c** APP/PS1 mice exhibited impaired spatial memory in the Morris water maze test. **d** Latency to reach the platform on the last day of training was higher in APP/PS1 mice. S14 treatment restored the spatial memory in APP/PS1 mice since latency to reach the platform was similar to wild-type mice. Data are expressed as mean ± SEM; *n* = 9–13 mice/group; *p < 0.05. Statistical significance was assessed by two-way ANOVA followed by Fisher’s post-hoc test for multiple comparisons. Veh vehicle

Behavioural tests were performed 28 days after oral drug administration following protocol A (Fig. 1a). An object recognition test was performed as described previously [27]. During this trial mice were placed individually in the open field in the presence of a familiar object, and the time spent exploring this object was measured. During a 10-min retention trial (second trial), which was performed 3 h later, a novel object was placed together with the familiar object in the open field. The time the animal spent exploring the two objects was recorded. The recognition index, defined as the ratio of the time spent exploring the novel object over the time spent exploring both objects, was used to measure non-spatial memory. Trials were recorded and tracked using the Smart Video Tracking System (Panlab). Spatial memory was tested using a Morris water maze for 4 consecutive days, carrying out four trials of memory training each day. Mice were allowed 60 s to reach the platform on the opposite side of the channel. Mice were allowed to stay on the platform for 15 s before being removed from the water. The latencies were determined by measuring the time to reach the platform. Twenty-four hours after the final trial, the platform was removed, and the mice were given probe trials to test their memory of the hidden platform. Trials were recorded and tracked using the Smart Video Tracking System (Panlab).

### Evaluation of neurogenesis

Neurogenic effects of orally administered S14 were evaluated after 5-bromo-2′-deoxyuridine (BrdU; 50 mg/kg) intraperitoneal injection once a day for 7 days, following protocol B (Fig. 1a). On day 35, 21 days after the last BrdU injection, the mice were anaesthetized with isoforane and perfused transcardially with 4% PFA. Fixed brains were cut on a vibratome (Leica Microsystems) at 40 mm, and tissue sections were collected in cold PB 0.1 M, and incubated overnight with primary antibodies at 4 °C. All primary antibodies were diluted in PB 0.1 M containing 0.5% bovine serum albumin and 0.5% Triton X-100. The following primary antibodies were used: rat monoclonal anti-BrdU (1:20,000; Hybridoma Bank), goat polyclonal anti-doublecortin (DCX; 1:500; Santa Cruz Biotechnology), and mouse monoclonal anti-neuronal nuclei (NeuN; 1:500; Millipore). After overnight incubation, primary antibody staining was revealed using the avidin-biotin complex method (VECTASTAIN Elite ABC Kit, Vector Laboratories, Burlingame, CA, USA) or fluorescence-conjugated secondary antibodies from Molecular Probes. To estimate the total number of BrdU^+^ cells in the brain, we performed 3,3’-diaminobenzidine (DAB) staining for BrdU on every sixth brain section. The number of BrdU^+^ cells in the granule cell of the dentate gyrus was counted using fluorescence microscopy (Zeiss microscope) at a magnification of 40× to estimate the total number of BrdU^+^ cells in the entire dentate gyrus. Based on a modified stereological method [28], BrdU^+^ were counted in one of every six sections from rostral (2 mm from the bregma) to caudal (−4.3 mm from the bregma). To determine the fate of dividing cells, 100–150 BrdU^+^ cells across 4–6 sections per mouse were analysed by fluorescence microscopy co-localizing with NeuN (NeuN^+^). The number of double-positive cells was expressed as a percentage of BrdU^+^ cells.

### cAMP assay

Measurement of cAMP levels was determined by enzyme-linked immunosorbent assay (ELISA) according to the manufacturer’s instructions (Arbor Assays) in brain tissue samples from APP/PS1 and wild-type mice.

### Immunoblot analysis

Proteins were isolated from brain tissue or cell cultures by standard methods. Briefly, brain tissues or cell lysates were homogenized in lysis buffer NP-40 (50 mM Tris-base, pH 7.4, 150 mM NaCl, 0.5% Nonidet P-40, 1 mM EDTA) containing a mixture of protease and phosphatase inhibitors (Roche Applied Science) and centrifuged for 15 min at 10,000 rpm at 4 °C. The supernatants were collected, and the total protein concentrations were measured by BCA assay (Pierce BCA Protein Assay Kit, Thermo Fisher, Waltham, MA, USA); proteins from each sample were loaded in a precast 4–20% Tris-Glycine gels (Novex) and transferred to polyvinylidene fluoride (PVDF) membranes (Bio-Rad). Primary antibodies used were: rabbit polyclonal anti-pCREB (1:500, Millipore), rabbit polyclonal anti-CREB (1:1000, Cell Signalling Technology), mouse anti-ATPasaβ (1:1000, Abcam), rabbit anti-PGC-1α (1:200, Santa Cruz), mouse anti-Mitofusin1 (Mfn1; 1:1000, Abcam), mouse anti-Mitofusin2 (Mfn2; 1:1000, Abcam), mouse anti-β actin horseradish peroxidase (HRP; 1:25,000, Abcam), mouse anti-mitochondrial dynamin-like GTPase (OPA1; 1:1000, Novus Biological), rabbit anti-Dynamin-related protein 1 (Drp1; 1:1000, Cell Signalling Technology), rabbit anti-p62/SQSTM1 (1:20,000, Abcam), and rabbit anti-light chain 3 (LC3; 1:1000, Novus Biological). Secondary HRP-conjugated goat anti-mouse (1:5000, Sigma) and goat anti-rabbit (1:5000, Life Technologies) were used. Immunoreactive bands were visualized by enhanced chemiluminescence (ECL; Pierce) and scanned on an ImageQuant LAS 4000 (GE Healthcare).

### Cell cultures

Human neuroblastoma (SH-SY5Y) cells were purchased from the European Collection of Cell Cultures (Health Protection Agency, Salisbury, UK) and maintained as previously described [29]. Unless otherwise stated, SH-SY5Y cells were seeded at a density of 4 × 10^4^ cells/cm^2^ and maintained in Dulbecco’s modified Eagle’s medium (DMEM) supplemented with 10% (v/v) fetal bovine serum (FBS) and 2 mM L-glutamine at 37 °C and 5% CO_2_. Cultures were incubated in fresh medium with or without S14 (15 μM), and alone or in combination with Aβ_42_ (1 μM). S14 was previously dissolved in dimethyl sulfoxide (DMSO) and then diluted in a sterile culture medium, as reported previously [30]. Aβ_42_ stock was previously dissolved in acetic acid 0.1 M. Oligomeric Aβ_42_ was prepared by incubating a volume of stock solution in DMEM at 4 °C for 24 h prior to addition to the cell cultures as previously described [31].

### Primary cortical neuronal cultures

Pregnant Wistar rats (3–5 months) were obtained from the inbred colony of the Research Institute, Hospital Doce de Octubre, Madrid, Spain. Primary cortical neuronal cultures were prepared from rat embryos (E15–16) based on methods previously described [32], and 4 × 10^5^ cells per well were plated on poly-l-lysine-coated coverslips in six-well plates. Cells were maintained at 37 °C in a 5% CO_2_ humidified atmosphere in Neurobasal A medium (Gibco) containing 2 mM l-glutamine, and 10% B27 Supplement (Invitrogen). Nine days after cell culture preparation, cortical neurons were treated with or without S14 (15 μM), alone or in combination with oligomerised Aβ_42_ (1 μM). All live cell imaging experiments were performed between days 10 and 14 in culture. All animals were handled and cared for according to the Council Directive 2010/63/UE of 22 September 2010.

### Mitochondrial mass assessment

To measure mitochondrial mass (mitochondrial volume as a percentage of cell volume in living cells) 40 min before the experiment, SH-SY5Y cells were loaded with Cell Trace Calcein-AM (Invitrogen) to visualise the whole cell volume and tetramethyl-rhodamine methylester (TMRM) to visualise the mitochondria inside each cell. High-resolution Z-stacks were acquired for approximately 15 cells per group using a confocal Zeiss 510 microscope equipped with a META detection system and a 63× oil immersion objective with an excitation/emission of 495/515 nm for calcein and 560/580 nm for TMRM. As TMRM is mitochondrial membrane potential dependent, the setting to detect red fluorescence was reduced to localise mitochondria still not degraded. Image analysis was carried out using *Volocity* software (PerkinElmer, Waltham, MA, USA) to measure the mitochondrial and cytosolic volume.

### Statistical analysis

Results are expressed as mean ± standard error of the mean (SEM). Statistical analyses were performed using a two-way analysis of variance (ANOVA) followed by Fisher’s post-hoc test for multiple comparisons. All calculations were made using GraphPad Prism6 software. Statistical significance was set at *p* < 0.05.

## Results

### Oral S14 treatment restored memory disturbances in APP/PS1 mice

Memory functioning was assessed in 6-month-old transgenic APP/PS1 and wild-type mice using two different behavioural tests. In the object recognition test, recognition index was significantly impaired in APP/PS1 mice compared to wild-type mice (*p* < 0.05; Fig. 1b). S14-treated APP/PS1 mice showed restoration of the impaired cognition similar to the wild-type group. Note that S14 administration in wild-type mice did not significantly alter behaviour. In the Morris water maze paradigm, APP/PS1 mice displayed significant spatial learning impairment compared with their littermate controls (*p* < 0.05; Fig. 1c), while S14-treated APP/PS1 mice were completely rescued from this impairment. Mice treated with this drug candidate displayed reductions in the latency to reach the hidden platform that was similar to those of wild-type controls during the probe test, suggesting that S14 induced an improvement in spatial memory (Fig. 1c). To further elucidate these differences, an additional analysis was conducted, comparing the latencies to reach the platform on the last day of training (Fig. 1d), demonstrating a significant overall difference among the groups (*p* < 0.05), with complete reversal of the memory impairment in S14-treated APP/PS1 mice (compared to wild-type mice).

### Oral S14 treatment enhanced hippocampal cell proliferation and neurogenesis in APP/PS1 mice

**Fig. 2:**
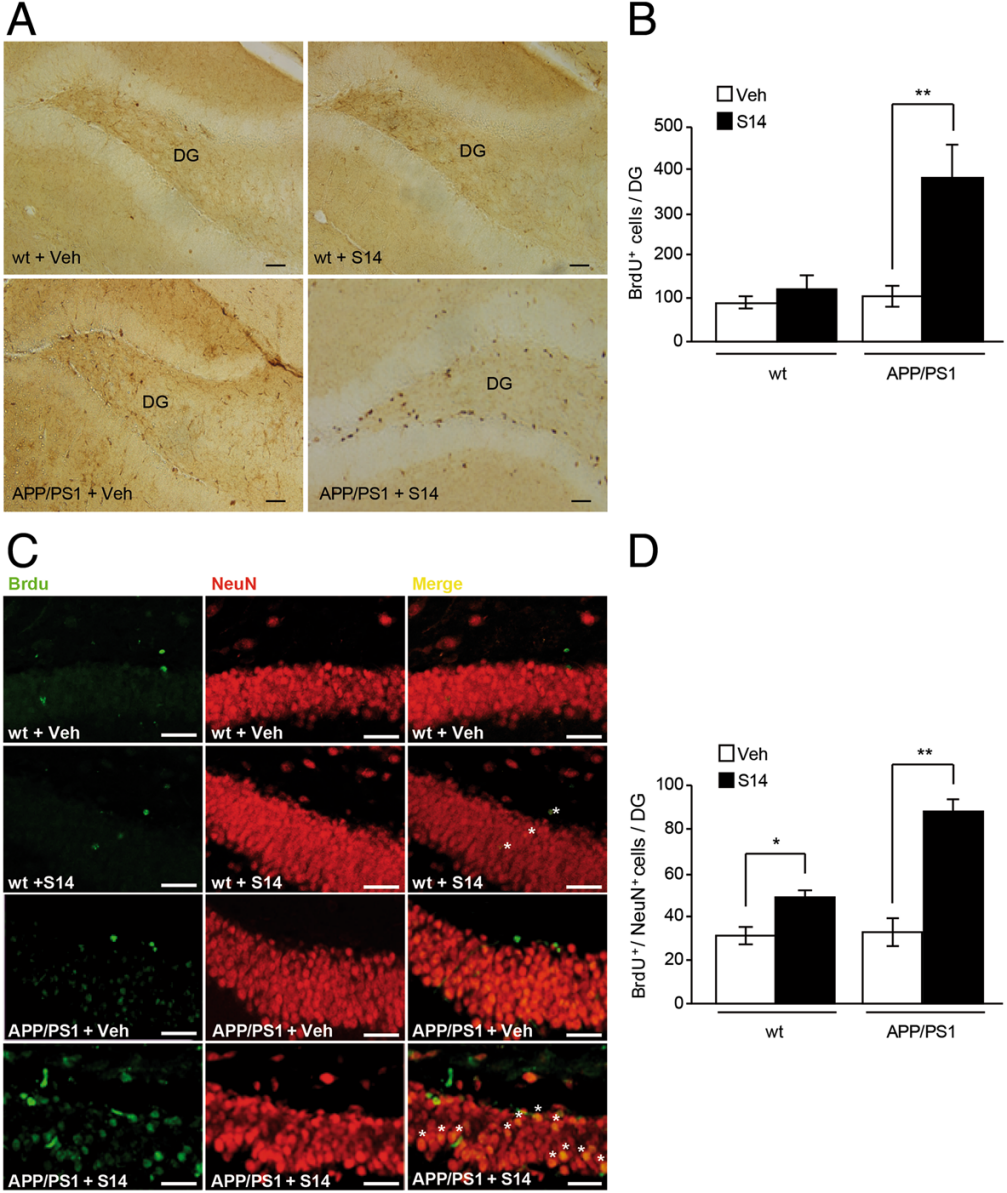
S14 treatment enhances hippocampal cell proliferation and neurogenesis in APP/PS1 mice. **a** Representative images showing 5-bromo-2’-deoxyuridine-positive (BrdU^+^) cells in the dentate gyrus (DG) in wild-type (wt) and APP/PS1 mice. Scale bar = 20 μm. **b** Quantification of BrdU^+^ cells showing higher cell proliferation in S14-treated APP/PS1 mice compared to vehicle (Veh)-treated mice. Data are expressed as mean ± SEM; *n* = 9–13 mice/group; **p < 0.01. **c** Representative images showing BrdU^+^/neuronal nuclei (NeuN)^+^ cells in the dentate gyrus in wild-type and APP/PS1 mice. Scale bar = 20 μm. **d** Quantification of BrdU^+^/NeuN^+^ cells showing a significant increase in neurogenesis in both S14-treated mice groups compared to vehicle-treated mice. Data are expressed as mean ± SEM; *n* = 9–13 mice/group; *p < 0.05; **p < 0.01. Statistical significance was assessed by two-way ANOVA followed by Fisher’s post-hoc test for multiple comparisons

The association between cognitive deterioration and decline in neurogenesis during aging, coupled with disruption in neurogenesis and cognitive dysfunction in AD mouse models, suggests that enhancing neurogenesis may be a feasible therapeutic approach. Thus, we explored the effects of S14 on hippocampal neurogenesis in mice sacrificed 21 days after the last BrdU injection. Positive BrdU cells (BrdU^+^) were predominantly localised in the sub-granular cell layer and, to a less extent, in the hilus (Fig. 2a). S14 treatment for 5 weeks significantly increased the number of BrdU^+^ cells in the dentate gyrus in APP/PS1 mice when compared with vehicle-treated mice (*p* < 0.01; Fig. 2b). Then the specific rate of new-born neurons in adult mice was evaluated in brain sections from S14- and vehicle-treated wild-type and APP/PS1 mice processed for dual-labelling immunohistochemistry with antibodies against BrdU and the neuronal-specific marker NeuN (Fig. 2c, d). Co-localisation analysis revealed that the number of BrdU^+^ neurons was significantly higher in both groups of S14-treated mice. Although the neurogenesis rate was found to be increased even in the wild-type group (*p* < 0.05), the neurogenesis was much higher in the APP/PS1-treated mice (*p* < 0.01; Fig. 2d).

### S14 treatment activates the hippocampal cAMP/pCREB pathway

**Fig. 3:**
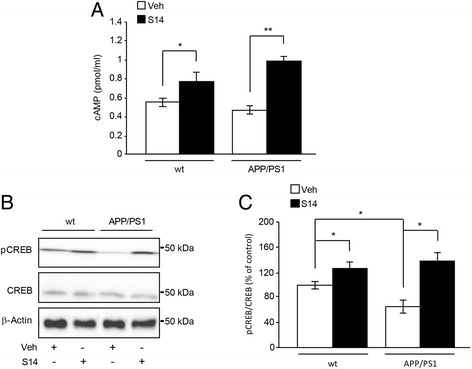
cAMP and pCREB levels in the hippocampus are increased after PDE7 inhibition in APP/PS1 and wild-type mice. **a** Cyclic adenosine monophosphate (cAMP) levels in the hippocampus of both S14-treated mice groups compared to untreated mice. Data are expressed as mean ± SEM; *n* = 6–7 mice/group; *p < 0.05; **p < 0.01. **b**,**c** Representative immunoblots and quantified levels of phosphorylated (p) and total cAMP-response element binding (CREB) protein. pCREB levels were reduced in the hippocampus of APP/PS1 mice, whereas S14 treatment increased pCREB expression in wild-type (wt) and APP/PS1 mice compared with vehicle-treated groups. Levels of total CREB remained unchanged. Data are expressed as mean ± SEM; *n* = 6–7 mice/group; *p < 0.05; **p < 0.01. Statistical significance was assessed by two-way ANOVA followed by Fisher’s post-hoc test for multiple comparisons. Veh vehicle

It is known PDE7 inhibitors activate the cAMP-dependent pathway [14, 33]; therefore, we examined the levels of cAMP and CREB activation (phospho-CREB, or pCREB) in the hippocampus from the different mice groups to probe the target engagement of S14 and the link with the observed higher rate of neurogenesis. We found that S14 treatment increased cAMP accumulation in both wild-type (*p* < 0.05; Fig. 3a) and APP/PS1 mice (*p* < 0.01; Fig. 3a), indicating that this compound reached both the target and the brain. A significant decrease in the phosphorylation status of CREB was found in the hippocampus of APP/PS1 mice compared to the wild-type group, showing no changes in the total CREB protein levels (*p* < 0.05; Fig. 3b, c). Conversely, S14 treatment increased the phosphorylation status of CREB in the hippocampus of both groups compared to vehicle-treated mice (Fig. 3b, c) showing no changes in the total CREB protein levels. As pCREB may be considered a surrogate biomarker of cAMP increasing, the enhancement of pCREB in the treated animals confirms that oral administration of S14 targets the cAMP-specific PDE7 pathway in the hippocampus.

### S14 treatment increases the PGC-1α levels and the mitochondrial biogenesis in the hippocampus of APP/PS1 mice and in Aβ-treated SH-SY5Y cells

**Fig. 4:**
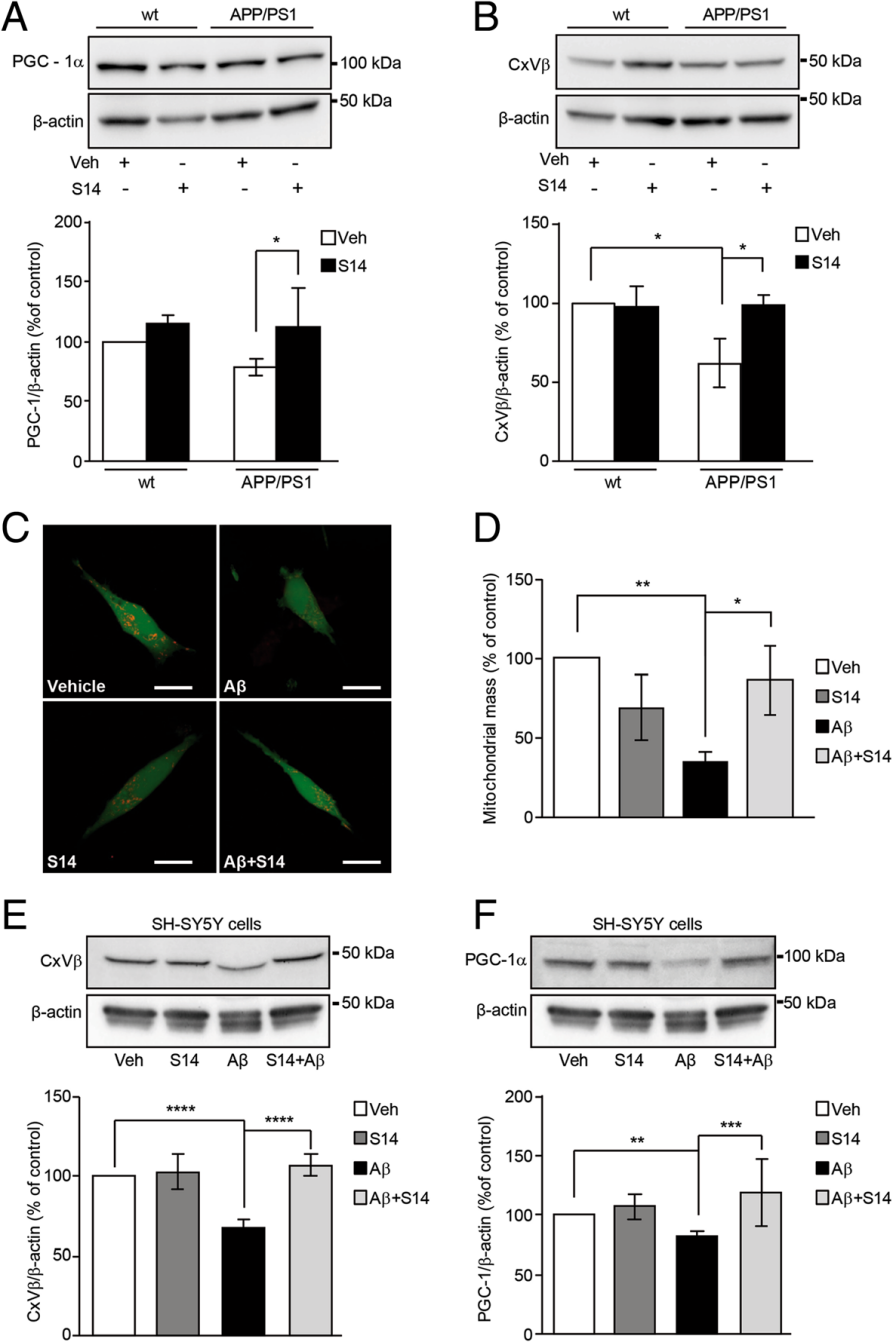
Amyloid β (Aβ)-induced reduction on mitochondrial biogenesis is restored after S14 treatment. **a** Upper panel shows representative Western blot bands, and the lower panel shows increased peroxisome proliferator-activated receptor γ co-activator α (PGC-1α) expression in the hippocampus of S14-treated APP/PS1 mice compared with the vehicle (Veh)-treated group (*n* = 5–7 mice per group; *p < 0.05). **b** Representative immunoblots (upper panels) and protein level quantification detected by Western blot (lower panel) showing a significant reduction in the expression of complex V β subunit (CxVβ) in the hippocampus of S14-treated APP/PS1 mice compared with the vehicle-treated group (*n* = 5–7 mice per group; *p < 0.05). When transgenic mice were treated with S14, CxVβ levels were recovered. Data are expressed as mean ± SEM. **c** Mitochondrial mass was measured in live cells using the human neuroblastome SH-SY5Y cell line simultaneously exposed to 1 μM oligomeric Aβ_42_ for 24 h and evaluated by confocal imaging. **d** Imaging analysis revealed a reduced amount of mitochondrial volume from the whole cell volume in Aβ_42_-exposed cells compared to untreated cells. Treatment with 15 μM S14 on Aβ_42_-exposed cells obliterated this effect, showing no changes on the unexposed cells. Data are expressed as mean ± SEM; *n* = 4; *p < 0.05; **p < 0.01. **e**,**f** Representative immunoblots (upper panel) and quantification of the measures detected by Western blot (lower panel) showing a significant reduction in the CxVβ levels (**e**) and PGC-1α (**f**) in SH-SY5Y cells exposed to 1 μM oligomeric Aβ_42._ When cells were co-treated with 15 μM S14, CxVβ (**e**) and PGC-1α (**f**) expression were recovered. Data are expressed as mean ± SEM; *n* = 4; **p < 0.01; ***p < 0.001; ****p < 0.0001. Statistical significance was assessed by two-way ANOVA followed by Fisher’s post-hoc test for multiple comparisons. wt wild-type

PGC-1α is a downstream effector of CREB activation. The levels of PGC-1α in the hippocampus of APP/PS1 mice tended to be reduced when compared to wild-type mice, but the difference was not statistically significant (Fig. 4a). S14 treatment, however, induced an increase in PGC-1α levels in APP/PS1 mice (Fig. 4a). PGC-1α is a master regulator of mitochondrial biogenesis and dynamics; therefore, we investigated the effect of S14 treatment on the mitochondrial biogenesis in wild-type and APP/PS1 mice in the hippocampus. To verify whether PGC-1α down- and upregulation by Aβ and S14, respectively, could cause changes in mitochondrial mass, we estimated the amount of total mitochondrial mass by analysing the levels of the representative mitochondrial protein complex V β subunit (CxVβ) using immunoblotting (Fig. 4b). Quantified Western blots revealed a significantly reduced amount of mitochondrial mass in APP/PS1 mice compared to the wild-type group (*p* < 0.05; Fig. 4b). S14 treatment restored the mitochondrial mass in APP/PS1 mice with no effects on wild-type mice (*p* < 0.05; Fig. 4b). Further evaluation of the effect of S14 on the mitochondrial mass was carried out in live cells using the human neuroblastoma SH-SY5Y cell line and rat primary cortical neurons. Cells were simultaneously exposed to 1 μM oligomeric Aβ_42_ for 24 h and mitochondrial mass was evaluated by confocal imaging (Fig. 4c, d, and Additional file 1: Figure S1). Imaging analysis revealed a reduced amount of mitochondrial volume from the whole cell volume in Aβ_42_-exposed cells compared to untreated cells (*p* < 0.01; Fig. 4d) in both SH-SY5Y cells and primary neuronal cultures. S14 treatment on Aβ_42_-exposed cells avoided this effect, showing no changes on those cells not exposed (*p* < 0.05; Fig. 4d and Additional file 1: Figure S1). Immunoblotting experiments on SH-SY5Y cells confirmed a reduced number of mitochondria, estimated by analysis of CxVβ levels, in SH-SY5Y cells exposed to oligomeric Aβ_42_ compared to untreated cells, and this effect was restored upon S14 treatment (*p* < 0.0001; Fig. 4e). Additionally, PGC-1α levels were found to be reduced in cells exposed to Aβ_42_ compared to untreated cells (*p* < 0.01), whereas co-administration with S14 completely abolished the Aβ_42_-induced effect (*p* < 0.001; Fig. 4f). In agreement with the above results, the PDE7 inhibitor S14 restored the damaged mitochondrial biogenesis due to Aβ overload as demonstrated in the APP/PS1 mice and SH-SY5Y cells.

### S14 treatment modulates Aβ-induced effects on mitochondrial fusion and fission processes

**Fig. 5:**
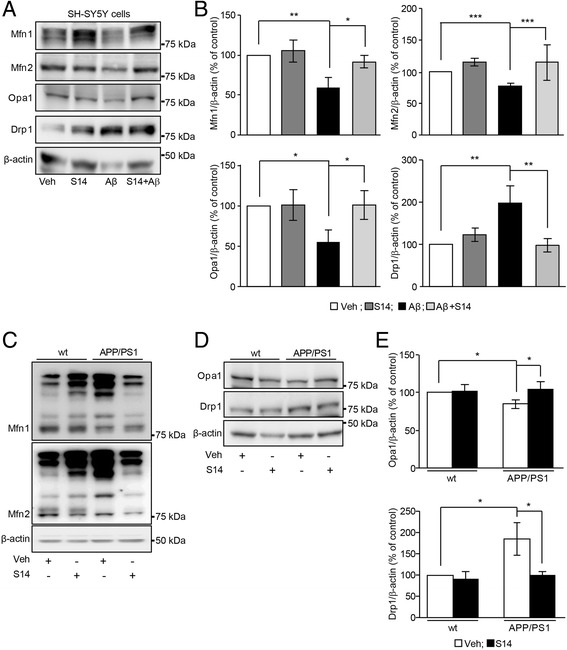
PDE7 inhibition modulates amyloid β (Aβ)-induced effects on mitochondrial fission, and fusion protein levels. **a** Immunoblots and **b** densitometric analysis of mitochondrial fission and fusion proteins in SH-SY5Y cells exposed to 1 μM oligomeric Aβ_42_. The fusion proteins mitofusin1 (Mfn1), mitofusin2 (Mfn2), and optic atrophy 1 (Opa1) were reduced in SH-SY5Y cells exposed to 1 μM oligomeric Aβ_42_. When cells were co-treated with S14, the expression of all these fusion proteins was recovered. Dynamin-related protein 1 (Drp1) levels were significantly increased in Aβ_42_-treated SH-SY5Y cells, and those levels were restored after co-administration of 15 μM S14. Data are expressed as mean ± SEM; *n* = 5–7; *p < 0.05; **p < 0.01; ***p < 0.001. **c** In the transgenic APP/PS1 mice, the ubiquitination pattern of mitofusins was enhanced compared to wild-type (wt) mice and S14 treatment abolished this ubiquitination. Whereas **d** Opa1 levels were reduced, **e** Drp1 expression was increased in the hippocampus of APP/PS1 and wild-type mice. S14 treatment restored these protein levels to those observed in wild-type mice. Data are expressed as mean ± SEM; *n* = 3–6 mice; *p < 0.05. Statistical significance was assessed by two-way ANOVA followed by Fisher’s post-hoc test for multiple comparisons. Veh vehicle

A combination of mitochondrial biogenesis, mitochondrial fusion/fission, and mitochondrial degradation is reflected in the amount and distribution of mitochondria in cells. As we have demonstrated, S14 can rescue the disrupted rate of mitochondrial biogenesis by Aβ overload we also answered whether PDE7 inhibition could induce changes in the fusion/fission mitochondrial quality control processes. Mitofusins 1 and 2 (Mfn1 and Mfn2, respectively) in the outer mitochondrial membrane and optic atrophy 1 (Opa1) in the inner mitochondrial membrane regulate the fusion process, and the dynamin-related protein 1 (Drp1) regulates the mitochondrial fission. A reduction in Mfn1, Mfn2, and Opa1 levels, as well as increased levels in Drp1, could indicate an increased rate of mitochondrial fission degradation. SH-SY5Y cells exposed to oligomeric Aβ_42_ showed reduced mitochondrial fusion proteins levels (Mfn1, Mfn2, and Opa1) and increased levels of the mitochondrial fission protein Drp1 (Fig. 5a, b). S14 treatment on SH-SY5Y cells exposed to Aβ_42_ rescued the levels of these proteins to values similar to those of the untreated cells (Fig. 5a, b). Upon mitochondrial damage, the outer mitochondrial membrane mitofusins are substrates for ubiquitination, increasing the mitochondrial fission rate. We therefore used the ubiquitination of Mfn1 and Mfn2 as a readout for activation of mitophagy in the hippocampus of the APP/PS1 mice. The ubiquitination pattern of mitofusins in transgenic mice was enhanced compared to wild-type mice (Fig. 5c). Accordingly, the levels of Opa1 were reduced and the Drp1 protein levels were found to be increased in APP/PS1 mice (*p* < 0.05; Fig. 5d, e). S14 treatment reduced the ubiquitination of Mfn1 and Mfn2 and significantly restored the Opa1 and Drp1 protein levels restoring them to wild-type values (*p* < 0.05; Fig. 5c–e). In summary, the PDE7 inhibitor S14 could rescue the mitochondrial fusion/fission balance previously impaired upon Aβ overload exposure.

### S14 treatment modulates Aβ-induced effects on mitophagy

**Fig. 6:**
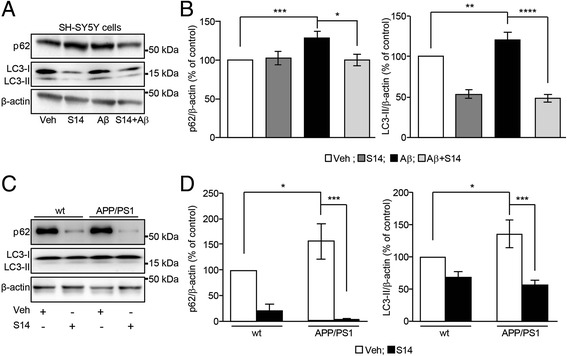
S14 treatment modulates amyloid β (Aβ)-induced effects on autophagic markers. **a** Representative Western blot and **b** estimated p62 and light chain 3 (LC3)-II protein levels in SH-SY5Y cells. Protein level analysis revealed a significant increase in p62 and LC3-II levels in SH-SY5Y cells exposed to 1 μM oligomeric Aβ_42_. When cells were co-treated with 15 μM S14, p62 and LC3-II levels were recovered. Data are expressed as mean ± SEM; *n* = 6; **p < 0.01; ***p < 0.001; ****p < 0.0001. **c** Representative Western blot and **d** estimated p62 and LC3-II protein levels in the hippocampus of APP/PS1 and wild-type (wt) mice. p62 and LC3-II protein levels were found increased in the hippocampus of APP/PS1 mice and S14 treatment was able to restore both to those observed in wild-type mice. Data are expressed as mean ± SEM; *n* = 3–7 mice; *p < 0.05; ***p < 0.001. Statistical significance was assessed by two-way ANOVA followed by Fisher’s post-hoc test for multiple comparisons. Veh vehicle

Ubiquitination of mitochondrial profusion proteins and increased levels of mitochondrial profission proteins may result in autophagic clearance of damaged mitochondria. Since the PDE7 inhibitor S14 rescued the Aβ-induced mitochondrial fusion/fission imbalance, we further investigate its effect on subsequent mitophagy. During induction of autophagy, LC3-I is lipidated on its mature form, LC3-II, which localizes to the autophagosome as it engulfs the substrate for degradation [34]. Immunoblotting revealed that Aβ_42_ exposure resulted in elevated LC3-II levels compared to untreated cells (*p* < 0.01; Fig. 6a, b). A similar effect was observed in APP/PS1 mice compared to the wild-type group (*p* < 0.05; Fig. 6c, d). S14 treatment in cells and APP/PS1 mice restored the LC3-II levels to similar values to untreated cells (*p* < 0.0001), and wild-type mice (*p* < 0.001), respectively. Another protein, p62, anchors both the exposed LC3-II in the phagophore (preformed autophagosome) and the damaged mitochondria prompted to be degraded; therefore, increased levels of p62 are also distinctive of autophagy. Immunoblotting carried out in SH-SY5Y cells showed that Aβ_42_ exposure induced a significant increase in p62 levels compared to untreated cells (*p* < 0.001; Fig. 6a, b), and a similar effect was found in the APP/PS1 mice compared to wild-type mice (*p* < 0.05; Fig. 6c, d). S14 treated cells co-exposed to Aβ_42_ exhibited similar levels of p62 to those from untreated cells (*p* < 0.001; Fig. 6a, b). PDE7 inhibitor treatment in mice induced a high reduction in the p62 levels in both wild-type and APP/PS1 mice, but the reduction in this was much higher in APP/PS1 mice whose p62 levels in the hippocampus were found to be 1.5-fold higher than in wild-type mice. This could be related to the increased cAMP observed in both wild-type and APP/PS1 mice since cAMP could upregulate p62 degradation (*p* < 0.001; Fig. 6c, d). In summary, the above results could indicate that S14 treatment abolished the Aβ-induced mitophagy by reducing the p62 levels and LC3 lipidation.

### PDE7 inhibition upregulates mitochondrial biogenesis and mitophagy in the cerebral cortex from APP/PS1 mice

**Fig. 7:**
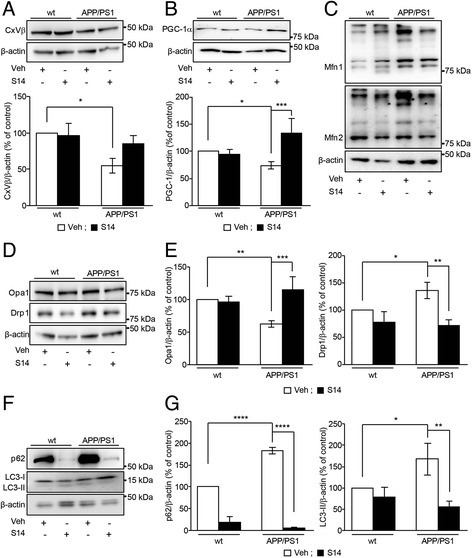
S14 treatment restores mitochondrial protein levels in the cerebral cortex in APP/PS1 mice. **a** Representative immunoblots (upper panels) and protein level quantification detected by Western blot (lower panel) showing a significant reduction in the complex V β subunit (CxVβ) levels in the cerebral cortex from APP/PS1 mice compared to the wild-type (wt) group. When transgenic mice were treated with S14, CxVβ levels were slightly but not significantly recovered (*n* = 3–5 mice per group; *p < 0.05). **b** Representative Western blot bands (upper panel), and quantified levels detected by Western blot (bottom panel) showing a significant reduction in Peroxisome proliferator-activated receptor γ co-activator α (PGC-1α) levels in the cerebral cortex from APP/PS1 mice compared with the wild-type group. When transgenic mice were treated with S14, PGC-1α levels were recovered. Data are expressed as mean ± SEM (*n* = 4–5 mice per group; *p < 0.05; ***p < 0.001). **c** Ubiquitination pattern of mitofusins in cerebral cortex from mice. S14 treatment restored the observed increased ubiquitination of mitofusin1 (Mfn1) and mitofusin2 (Mfn2) in the APP/PS1 transgenic mice. **d** Representative Western blot and **e** estimated optic atrophy 1 (Opa1) and dynamin-related protein 1 (Drp1) protein levels in the cortex from APP/PS1 and wild-type mice. Opa1 protein levels were found to be reduced and Drp1 levels were found to be increased in APP/PS1 mice. S14 treatment was able to restore both to those observed in wild-type mice. Data are expressed as mean ± SEM; *n* = 3–6 mice per group; *p < 0.05; ***p < 0.001. **f** Representative Western blot and **g** estimated p62 and light chain 3 (LC3)-II protein levels in the cortex from APP/PS1 and wild-type mice. p62 and LC3-II protein levels were found to be increased in the cerebral cortex from APP/PS1 mice, and S14 treatment was able to restore both to those observed in wild-type mice. Data are expressed as mean ± SEM; *n* = 3–5 mice per group; *p < 0.05; **p < 0.01; ****p < 0.0001. Statistical significance was assessed by two-way ANOVA followed by Fisher’s post-hoc test for multiple comparisons. Veh vehicle

Recognition memory control is typically attributed to the hippocampus, but the cerebral cortex is also controlling this process. Therefore, we investigated if S14 could interfere in mitochondrial dynamic alterations in the same way as it is interfering in the hippocampus. We confirmed that S14 also induced a twofold increase in cAMP levels in cortical samples from APP/PS1 (untreated APP/PS1 = 0.61 pmol/ml; APP/PS1 + S14 = 1.13 pmol/ml; *n* = 4 mice/group). The analysis of CxVβ protein levels revealed reduced mitochondrial mass in cortical lysates from APP/PS1 mice compared to wild-type mice (Fig. 7a). The reduction in the mitochondrial mass in APP/PS1 mice was subsequently related to a reduction in the mitochondrial biogenesis reflected in reduced PGC-1α levels compared to the wild-type group (*p* < 0.05; Fig. 7b) and imbalances in the mitochondrial fusion/fission events as demonstrated by increased ubiquitination of Mfn1 and Mfn2, reduced levels of Opa1 (*p* < 0.01), and increased levels of Drp1 (*p* < 0.05) in APP/PS1 mice compared to wild-type mice (Fig. 7c–e). S14 treatment did not significantly rescue the reduced mitochondrial mass in the cerebral cortex from APP/PS1 mice; however, the PGC-1α levels, the ubiquitination pattern of Mfn1 and Mfn2, and the Opa1 and Drp1 levels in APP/PS1 mice were significantly restored to equivalent values to the wild-type group (Fig. 7a–e). Equivalent to the effect observed in hippocampus, S14 treatment significantly reduced the p62 levels in both wild-type and APP/PS1 mice but this reduction was much more significant in APP/PS1 mice whose p62 levels in the cortex were found to be 1.8-fold higher than wild-type mice (Fig. 7f). The LC3-I lipidation to LC3-II was found to be increased in the cortex from APP/PS1 mice (*p* < 0.05) compared to wild-type mice, and S14 treatment attenuated this effect in APP/PS1 mice (Fig. 7g). The above results may indicate that the PDE7 inhibitor S14 could ameliorate the behavioural impairments induced by Aβ overload in the cerebral cortex of the APP/PS1 mice by interfering in the mitochondrial dynamics.

## Discussion

n this study, we investigated the effects of oral administration of the small heterocyclic molecule S14 on the neuronal and cognitive features of AD. We report here that inhibition of PDE7 reversed hippocampal and cortical Aβ-induced memory impairments, restored CREB phosphorylation via increasing intracellular cAMP levels, and increased the hippocampal neurogenesis in transgenic APP/PS1 mice. We also find that co-administration of S14 abolished the Aβ-induced mitochondrial dynamic impairments.

Our results showed that S14 was able to restore learning and memory deficits in APP/PS1 mice as shown by a reduced latency to reach the hidden platform in the Morris water maze. Consistent with our previous studies using intraperitoneal administration [14], oral treatment with S14 also improved non-spatial visual recognition memory measured in the object recognition test. Inhibitors of PDE7 have proven to be effective experimental drugs for exploring cognitive and motor recovery [14, 35–37].

We provide several new insights into the mechanisms of S14-enhanced hippocampal neurogenesis and memory in APP/PS1 mice. The hippocampus is a key brain structure for learning and memory and one of the main brain regions to be affected in AD, which arises on the pathological background of Aβ burden, neurofibrillary tangles, and synaptic and neuronal loss [38]. Although decreased neurogenesis is generally thought to be responsible for learning and memory impairments in AD, it is somewhat controversial whether neurogenesis is decreased or increased in the context of AD [39–42]. Here, we show that neurogenesis was not significantly different in the dentate gyrus of 6-month-old APP/PS1 mice compared to the wild-type group as assessed by BrdU^+^/NeuN^+^ staining. However, S14 treatment was found to upregulate neurogenesis in the dentate gyrus of these animals; this was much more increased in the transgenic mice. The observed memory improvements in APP/PS1 mice may be related to the subsequent increase in intracellular cAMP and pCREB levels that we found in the cortex and hippocampus after PDE7 inhibition using S14. CREB is a key regulator of differentiation and maturation in adult neurogenesis [43] and its activation by phosphorylation leads to transcription of memory-associated genes as a hub of pathways and mechanisms activated during the processes of synaptic strengthening and memory formation [44]. Disruption of these mechanisms in AD results in reduced CREB activation and memory impairments [45]. Decreased activation of CREB in our APP/PS1 mice and its re-establishment after chronic oral treatment with S14 in the cortex and hippocampus suggest that the cAMP/CREB cascade may be involved in the protective role of S14 on synaptic strengthening and memory improvement/enhancement/long-term potentiation (LTP) memory, in agreement with new therapeutic strategies based on the use of drugs enhancing CREB activation [46].

Our findings show that S14 treatment rescued the reduced mitochondrial mass in both Aβ-treated SH-SY5Y cells and APP/PS1 mice. Reduced mitochondrial mass has already been found in brains from AD patients compared to healthy controls in a mouse model of AD and in AD cellular models [47–49]. A reduction in the number of mitochondria could be a consequence of impairments in the mitochondrial biogenesis or enhanced mitochondrial-specific autophagy clearance known as mitophagy. Activation of the cAMP/CREB pathway resulting in the phosphorylation of CREB contributes to enhance the transcription of the PGC-1α promoter, a key regulator of mitochondrial biogenesis [19, 20]. Our data showed that S14 treatment increased PGC-1α levels, reversing the Aβ-mediated reduction in mitochondrial biogenesis. Aβ-induced decreased mitochondrial biogenesis has been found previously by other authors [50, 51]. Additional studies show that mitochondrial DNA copy number and the expression levels of PGC-1α were significantly reduced in brains from AD patients and transgenic mouse models of AD, suggesting impaired mitochondrial biogenesis [23, 52–55]. Mitochondria are essential in adult neurogenesis, including having a modulatory function over maturation of adult-born hippocampal neurons [56]. Transcriptional pathways may not only control the expression of neuron-specific genes but may also target mitochondrial biogenesis. In this regard, the transcription factor CREB may fulfil coordinative functions given that its target, PGC-1α, has been linked with the control of cell maturation in hippocampal neurons [57]. It is reasonable that S14 treatment, by improving neurogenesis in APP/PS1 mice, simultaneously induces mitochondrial biogenesis since mitochondria are the power houses of neurons, providing energy for cytoskeletal remodelling, outgrowth of neuronal processes, growth cone activity, and synaptic activity. The number of mitochondria may also be controlled by mitophagy, a tightly regulated process in which damaged or non-functional mitochondria are separated from the mitochondrial network and prompted for degradation. We showed that Aβ treatment shifted the fusion/fission balance towards increased fission. The PDE7 inhibitor S14 restored the Aβ-reduced levels of the mitochondrial profusion proteins Mfn1, Mfn2, and Opa1, and, conversely, it rescued the Aβ-increased levels of the fission protein Drp1. Aβ treatment shifted the fusion/fission balance towards increased fission. A balance between the mitochondrial levels of these fusion/fission proteins is essential to maintain a functional mitochondrial network in the cells. This has been further demonstrated in brains from AD patients and mouse and cellular models of AD related to Aβ deposition [58, 59]. Based on the Aβ accumulation effect, our results link PDE7 inhibition and downstream signalling with a restoration of the fusion/fission protein levels. Additionally, Mfn2, whose expression is induced by PGC-1α, regulates not only the mitochondrial fusion but also mitochondrial biogenesis and mitochondrial function through changes in mitochondrial membrane potential and the expression of OXPHOS subunits [60].

Finally, we have demonstrated PDE7 inhibition abolished the Aβ-induced mitophagy by reduction in LC3-II and p62 levels previously increased as a consequence of misbalances on mitochondrial fusion/fission events after Aβ exposure/overload. Mitofusin ubiquitination induces their proteasomal degradation and subsequently reduces the mitofusin levels. This, along with reduced Opa1 and increased Drp1 levels, triggers the split of damaged mitochondria from the mitochondrial network. At the same time, ubiquitination of mitochondrial proteins targets the mitochondria to be engulfed by autophagosome membranes showing increased lipidated LC3 (LC3-II) levels. p62 protein anchors the targeted mitochondria to the autophagosome membranes. Previous work has shown reduced autophagy rates in several disease models by PDE inhibition. In particular, p62 degradation has been found to be increased in several works analysing the effects of antioxidants on autophagy [17, 21]. In these studies, authors reported increased levels of cAMP regulating the increased degradation of p62, thus reducing p62 levels. In 2015, Boland et al. simultaneously inhibited most PDE isoforms targeting the cAMP/CREB pathway by using classical inhibitors of autophagy [61]. They observed that this treatment increased the cAMP in pancreatic cells, increasing the insulin production that was formerly deteriorated [61]. In other work, Hosokawa et al. reported that PDE5 inhibition restored the increased autophagy rates in a muscle weakness mouse model [62]. We can conclude that S14 treatment reduces the previously Aβ-increased mitophagy rates. It is known that Aβ induces mitophagy, as has been demonstrated in recent studies [58, 59]. We can speculate that PDE7 inhibition through S14 can regulate the mitochondrial homeostasis by increasing the cAMP levels, as has been recently reviewed by Di Benedetto et al. [63]. Since mitochondrial dysfunction is involved in many neurodegenerative diseases and aging [64], S14 may slow down pathological events in the neurodegenerative process.

## Conclusions

In conclusion, our data provide evidence that PDE7 inhibition improved hippocampal neurogenesis and memory by modulating cAMP/pCREB activity and that these effects may be associated with mitochondrial dynamics regulation. Considering that S14 treatment has been reported to be beneficial for AD [14] and other neurodegenerative disorders such as Parkinson’s disease [15, 30, 37], this study suggests that S14 is a very promising disease-modifying drug candidate for the future treatment of neurodegenerative disorders.

## Additional file

Additional file 1 **Figure S1.** Aβ-induced reduction in mitochondrial biogenesis in neurons is restored after S14 treatment. Mitochondrial mass was carried out in live rat neuronal primary cultures simultaneously exposed to 1 μM oligomeric Aβ_42_ for 24 h and evaluated by confocal imaging. Representative images showing the mitochondria and the whole cell body with the range of treatments are presented in the left panels. Imaging analysis revealed a reduced amount of mitochondrial volume from the whole cell volume in Aβ_42_-exposed cells compared to untreated cells. Treatment with 15 μM S14 on Aβ_42_-exposed cells obliterated this effect, showing no changes in the unexposed cells. Statistical significance was assessed by two-way ANOVA followed by Fisher’s post-hoc test for multiple comparisons. Data are expressed as mean ± SEM; *n* = 4; **p < 0.01; ****p < 0.0001. (JPEG 1623 kb)

## Abbreviations

Aβ: Amyloid β
AD: Alzheimer’s disease
APP/PS1: APP_SWE_/PS1_M146L_
BrdU: 5-Bromo-2′-deoxyuridine
cAMP: Cyclic adenosine monophosphate
*CREB*: cAMP-response element binding protein
CxVβ: Complex V β subunit
DAB: 3,3′-Diaminobenzidine
DCX: Doublecortin
DMEM: Dulbecco’s modified Eagle’s medium
Drp1: Dynamin-related protein 1
FBS: Fetal bovine serum
HRP: Horseradish peroxidase
LC3: Light chain 3
Mfn1: Mitofusin1
Mfn2: Mitofusin2
NeuN: Neuronal nuclei
Opa1: Optic atrophy 1
PB: Phosphate buffer
PDE: Phosphodiesterase
PFA: Paraformaldehyde
PGC-1α: Peroxisome proliferator-activated receptor γ co-activator α
TMRM: Tetramethyl-rhodamine methylester
