# Supplementary figures and images for: Amyloid β-induced impairments on mitochondrial dynamics, hippocampal neurogenesis, and memory are restored by phosphodiesterase 7 inhibition
Source: Alzheimers Res Ther. 2018 Feb 20;10:24. doi: 10.1186/s13195-018-0352-4 (PMC5819290; doi:10.1186/s13195-018-0352-4)

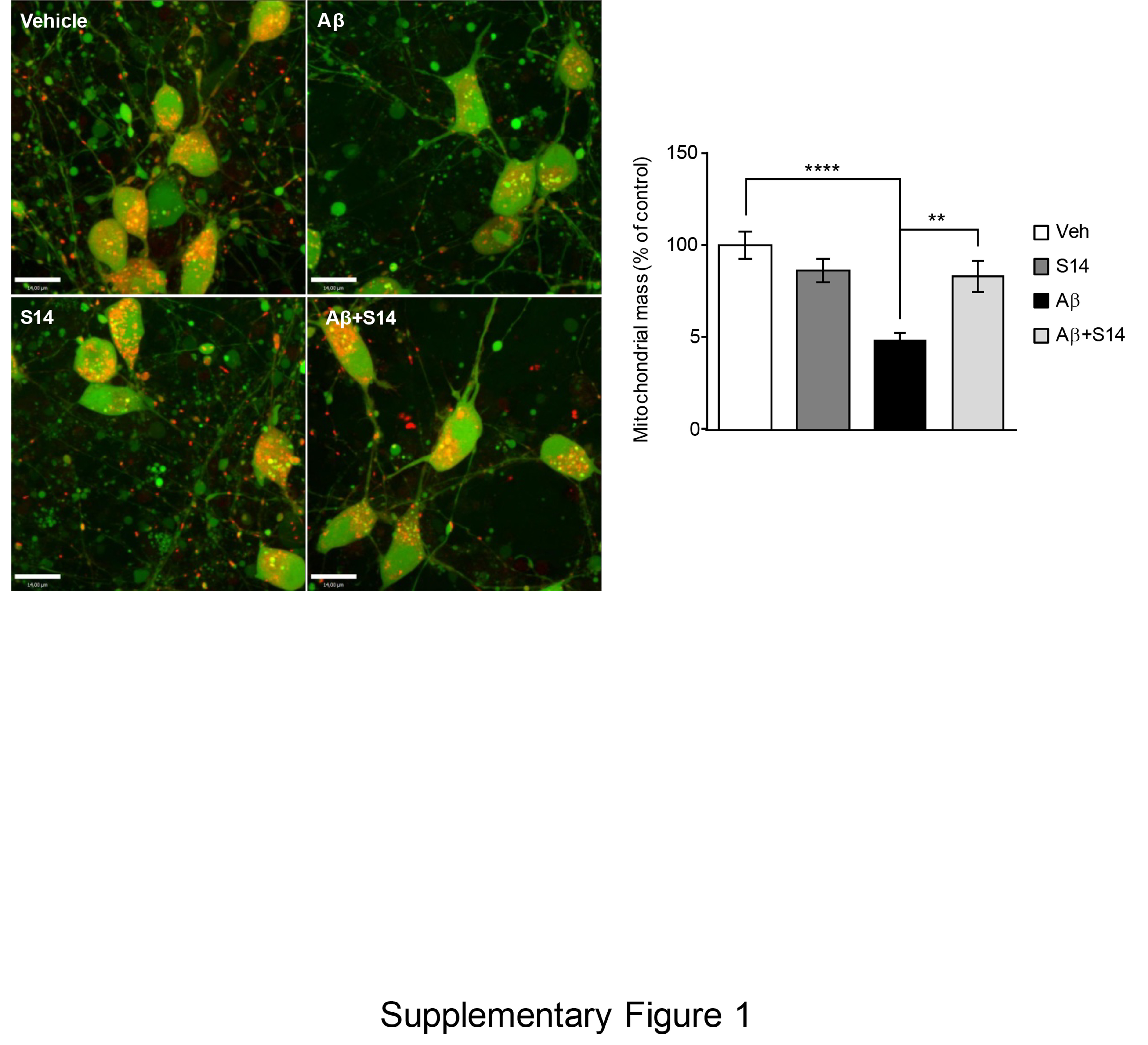

Supplement: Supplementary file 1 — Figure S1. Aβ-induced reduction in mitochondrial biogenesis in neurons is restored after S14 treatment. Mitochondrial mass was carried out in live rat neuronal primary cultures simultaneously exposed to 1 μM oligomeric Aβ42 for 24 h and evaluated by confocal imaging. Representative images showing the mitochondria and the whole cell body with the range of treatments are presented in the left panels. Imaging analysis revealed a reduced amount of mitochondrial volume from the whole cell volume in Aβ42-exposed cells compared to untreated cells. Treatment with 15 μM S14 on Aβ42-exposed cells obliterated this effect, showing no changes in the unexposed cells. Statistical significance was assessed by two-way ANOVA followed by Fisher’s post-hoc test for multiple comparisons. Data are expressed as mean ± SEM; n = 4; **p < 0.01; ****p < 0.0001. (JPEG 1623 kb) [file 13195_2018_352_MOESM1_ESM.jpg]
